# From Cerebellar Activation and Connectivity to Cognition: A Review of the Quadrato Motor Training

**DOI:** 10.1155/2015/954901

**Published:** 2015-10-11

**Authors:** Tal Dotan Ben-Soussan, Joseph Glicksohn, Aviva Berkovich-Ohana

**Affiliations:** ^1^The Leslie and Susan Gonda (Goldschmied) Multidisciplinary Brain Research Center, Bar-Ilan University, Ramat Gan, Israel; ^2^Research Institute for Neuroscience, Education and Didactics, Patrizio Paoletti Foundation, Rome, Italy; ^3^Department of Criminology, Bar-Ilan University, Ramat Gan, Israel; ^4^Department of Neurobiology, Weizmann Institute of Science, Rehovot, Israel; ^5^Department of Physiology and Pharmacology, University of Rome “La Sapienza”, Rome, Italy

## Abstract

The importance of the cerebellum is increasingly recognized, not only in motor control but also in cognitive learning and function. Nevertheless, the relationship between training-induced cerebellar activation and electrophysiological and structural changes in humans has yet to be established. In the current paper, we suggest a general model tying cerebellar function to cognitive improvement, via neuronal synchronization, as well as biochemical and anatomical changes. We then suggest that sensorimotor training provides an optimal paradigm to test the proposed model and review supporting evidence of Quadrato Motor Training (QMT), a sensorimotor training aimed at increasing attention and coordination. Subsequently, we discuss the possible mechanisms through which QMT may exert its beneficial effects on cognition (e.g., increased creativity, reflectivity, and reading), focusing on cerebellar alpha activity as a possible mediating mechanism allowing cognitive improvement, molecular and anatomical changes. Using the example of QMT research, this paper emphasizes the importance of investigating whole-body sensorimotor training paradigms utilizing a multidisciplinary approach and its implications to healthy brain development.

## 1. Introduction

In this paper, we suggest a general model tying cerebellum function to cognitive improvement, via neuronal synchronization and multimodal connectivity. We then discuss sensorimotor training effects in humans, specifically the Quadrato Motor Training (QMT) effects on brain connectivity, cognitive function, and structural and molecular changes, supporting such a multimodal cerebellar-cognition relationship.

While the role of the cerebellum was traditionally acknowledged in the field of motor control [1–4], it is increasingly recognized in the last decade in sensory regulation and cognitive learning [5–7]. Particularly, accumulating evidence suggests that the cerebellum serves as a general timing mechanism for both sensorimotor and cognitive processes [8–10]. This may be conducted by the cerebellum's role in regulating the rate, force, rhythm, and accuracy of movements, which are crucial for controlling the speed, capacity, consistency, and appropriateness of cognitive as well as emotional processes [6, 11, 12]. In fact, motor learning studies have long been aware of the cerebellum's oscillatory role in skill acquisition, such as bimanual ability [13–15].

Consistent with its putative role in cognition, the cerebellum forms close connections with neocortical brain regions including the prefrontal cortex, thus providing the neural basis through which the cerebellum contributes to neocortical information processing (for review see [16]). Importantly, both the cerebellum and the prefrontal cortex play a crucial role in the neural network, which is activated when attention engagement or shifting are required, especially during challenging as well as novel tasks [7]. Indeed, a recent local field potential study has demonstrated the directionality of low frequencies (5–10 Hz) coherence as directed from the cerebellum to frontal areas, playing a role in goal-directed motion [16]. The fact that these anatomical connections are crucial for cognitive development and functions further highlights the importance of moving from the research focusing solely on corticocortical connections to an integrated examination of subcortical regions, and especially the cerebellum [7, 17–19]. The cerebellum has been studied mainly by using animal models (e.g., [20]).

Animals models demonstrate that cerebellar-related deficits are accompanied by cognitive impairment (e.g., memory, attention, and visuospatial abilities), as well as cerebellar microstructural changes as a result of training and exposure to enriched environment (for reviews see [21, 22]). In humans, it is widely accepted that only after the introduction of positron emission tomography (PET) and functional magnetic resonance imaging (fMRI) did it become possible to detect cerebellar activation noninvasively during natural movements (reviewed by [23]). FMRI studies report, for example, cerebellar activation during verbal working memory and finger-tapping tasks [24–26].

While electroencephalographic (EEG) studies have generally avoided studying cerebellar function, due to the fact that the neurons in the cerebellum are arranged in a “closed field” configuration [27], the invention of magnetoencephalography (MEG) provides another possible imaging tool, as electrophysiological signals can be obtained by MEG from the cerebellum, especially the slow rhythms including theta (4–7 Hz) and alpha (8–12 Hz) range [10, 28, 29]. For animal models explaining the possible underlying mechanisms resulting in cerebellar low rhythm oscillations, see [30, 31]. For example, cerebellar activity before a stimulus onset predicts uncued simple reaction time, possibly reflecting cerebellar role in timing, response readiness, prediction, and attention [32]. Thus, new neuroimaging methods in the last 2 decades enabled the noninvasive investigation of the cerebellum in humans. These lines of studies consistently have shown the involvement of the cerebellum in many cognitive functions, and specifically in language and reading [33, 34]. The above accumulating studies support the importance of cerebellar activity and connectivity for higher cognitive functions, including timing, attention, emotion, and cognition.

**Figure 1:**
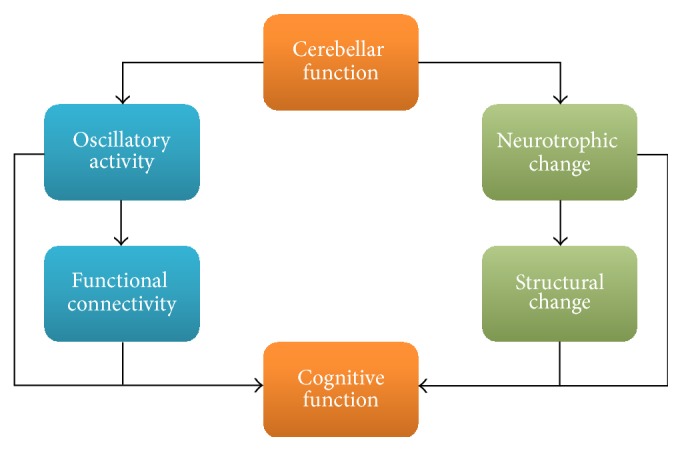
Interconnected relationship between cerebellum and cognitive function. The relationship is mediated via two interrelated routes. The first is slow rhythm oscillations, manifested in functional connectivity. The second is molecular effects on structural changes in structural connectivity.

In the model we present here, the importance of the cerebellar oscillatory activity for higher cognitive function is emphasized possibly mediated through several levels of brain organization, including molecular, anatomical, and neural oscillations (Figure 1). Our model suggests that the importance of cerebellar function for cognition may be based at least partly on two interrelated pathways, the first being alterations in oscillatory activity, leading to changes in functional connectivity [35–37], and the second which takes longer periods to occur is mediated by neurotrophic factors, leading to anatomical changes.

The suggested model is based on the following considerations.

*(1) Electrophysiological Mechanisms Mediating Cerebellar-Cognition Relationship*. Cerebellar alpha oscillatory activity has been suggested to be the neural system underlying the planning and execution of movements as well as reading and language comprehension [36–38]. The cerebellum was further found to be the main alpha source of the cerebrocerebellar network that is crucial for speech production, visual recognition and working memory, and coordination [38]. Thus, cerebellar alpha oscillations may mediate both cerebrocerebellar as well as cortical communication, such as the frontoparietal network [35, 38]. This is further in line with previous studies suggesting that sensorimotor-dependent cerebellar stimulation may modulate ongoing firing patterns in frontal regions that are crucial for cognitive functions, such as planning and creativity [16, 39, 40].

*(2) Anatomical and Molecular Mechanisms Mediating Cerebellar-Cognition Relationship*. Our model focuses on BDNF and Nerve Growth Factor (NGF), which are related to learning and neural plasticity. Neurotrophins, initially synthesized as precursor proteins (proneurotrophins), can influence both developing and mature neural circuits, utilizing distinct receptors to mediate divergent neuronal actions [41, 42]. Thus, while proBDNF and NGF were shown to be related to learning, spatial cognition, and neuronal plasticity [43–46], proNGF was reported to play an important role in nociceptors, neuronal death, and neurodegeneration [47]. Importantly, BDNF is considered a key component mediating neuronal connectivity and movement dependent plasticity especially in the cerebellum [48, 49]. More specifically, motor training is accompanied by increased cerebellar BDNF mRNA amount [50, 51] and structural changes related to underlying cellular events, including synaptogenesis and dendritic arborisation [49, 52]. On the other hand, animal models have shown that severe deficiencies in motor coordination in BDNF knockout mice are linked to abnormal cerebellar development [53, 54]. In addition, sensorimotor training-induced functional connectivity changes were found to be BDNF-dependent [55, 56].

Thus, we further present the possible effect induced by motor interventions in humans on the putative cerebellar-cognition relationship. Given the importance of the cerebellum in motor control, surprisingly few studies focused on cerebellar motor training effects in humans. In contrast, numerous animal models have consistently demonstrated training-induced effects on brain regions involved in locomotion such as the motor cortex, the basal ganglia, and the cerebellum (for review see [21, 22, 55, 56]). Nevertheless, human studies examining whole-body training-induced changes in executive functions have generally neglected motor-related areas. These studies have almost exclusively concentrated on frontal effects, generally reporting increased frontal alpha synchronization following training [39, 40, 57].

Interestingly, previous studies have found different procedures, such as transcranial direct current stimulation, to improve motor skill learning through augmentation of synaptic plasticity that requires BDNF-secretion [46]. Yet, noninvasive techniques, such as sensorimotor training paradigms, may serve as time and cost-efficient means of reaching similar effects, with no side effects. In line with this, while animal models of pharmacological treatment aimed at cerebellar compensation is largely unsatisfactory, exercise has been found to have some efficacy (for review see [21, 22]). For example, animal models of active training and enriched environment [58–60], as well as a few human studies [61, 62] have demonstrated cerebellar microstructural changes following sensorimotor training.

Consequently, we suggest that motor training in humans may serve as an optimal model to test the possible cerebellar-cognition relationship in two interrelated routes: (1) sensorimotor training may result in fast occurring, cerebellar and frontal low frequency oscillatory modulation [16], possibly leading to improved cognitive performance [40, 63]. (2) Changes in cerebellar function can be further stimulated through training by activating molecular mechanisms, mainly through the regulation of neurotrophins [48, 51].

Consequently, we review a series of sensorimotor training studies aimed at increasing our understanding of the possible link between cerebellar-induced change and cognitive improvement. This review will assist in supporting our presented model (Figure 1), providing evidence for training-induced alterations in alpha oscillation, anatomical, molecular, and cognitive change as a result of a specific sensorimotor training. The training method employed is the* Quadrato Motor Training* (QMT), a sensorimotor whole-body training.

## 2. Putting Theory into Practice: The Case of QMT

**Figure 2:**
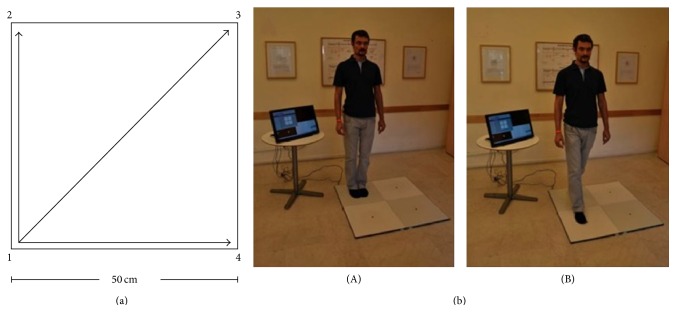
The Quadrato Motor Training (QMT). (a) A graphical illustration of the QMT. (b) A participant during the QMT while waiting for the next instruction (A) and following the instruction (B). Adapted from [64].

QMT is a new method for motor training, developed by Patrizio Paoletti, generally aiming at enhancing coordination, attention, and creativity [64, 65]. QMT involves following a structured set of simple oral instructions, by stepping to the instructed corner in a 50 × 50 cm square. See Figure 2.

The QMT requires a state of enhanced attention, as it combines dividing attention to the motor response and cognitive processing for producing the correct direction of movement to the next point in the Quadrato space [65, 70]. The QMT has the advantage of being a relatively short training (possibly several minutes) and can be relatively easily practiced in limited spaces. In addition, in comparison to other whole-body training paradigms, it can easily be quantified in terms of accuracy and reaction time. Together these unique aspects render the QMT a technique warranted of scientific exploration, with the future aim of implementing this technique in various health-promoting and educational setups. Next, we review results related to short-term effects of the QMT, that is, one practice of several minutes, as well as long-term effects, following a period of one-three months, in order to add several steps towards verifying our hypothesis.

### 2.1. Short-Term QMT-Induced Effects

Deficits in spatial cognition, creativity, and decreased cerebellar activity are reported in different developmental disorders and neurodegenerative diseases [71–74], emphasizing the importance of cerebellar oscillatory activity [1, 10, 13, 14, 75, 76]. While we could not study cerebellar electrophysiology directly, we studied the effect of one QMT session on low rhythm oscillations and connectivity, thought to mediate cerebellar function [66] and several cognitive skills, including creativity, spatial performance, reaction time, and reflectivity [64–68, 70].

#### 2.1.1. Creativity, Reaction Time, Alpha Power, and Coherence

**Figure 3:**
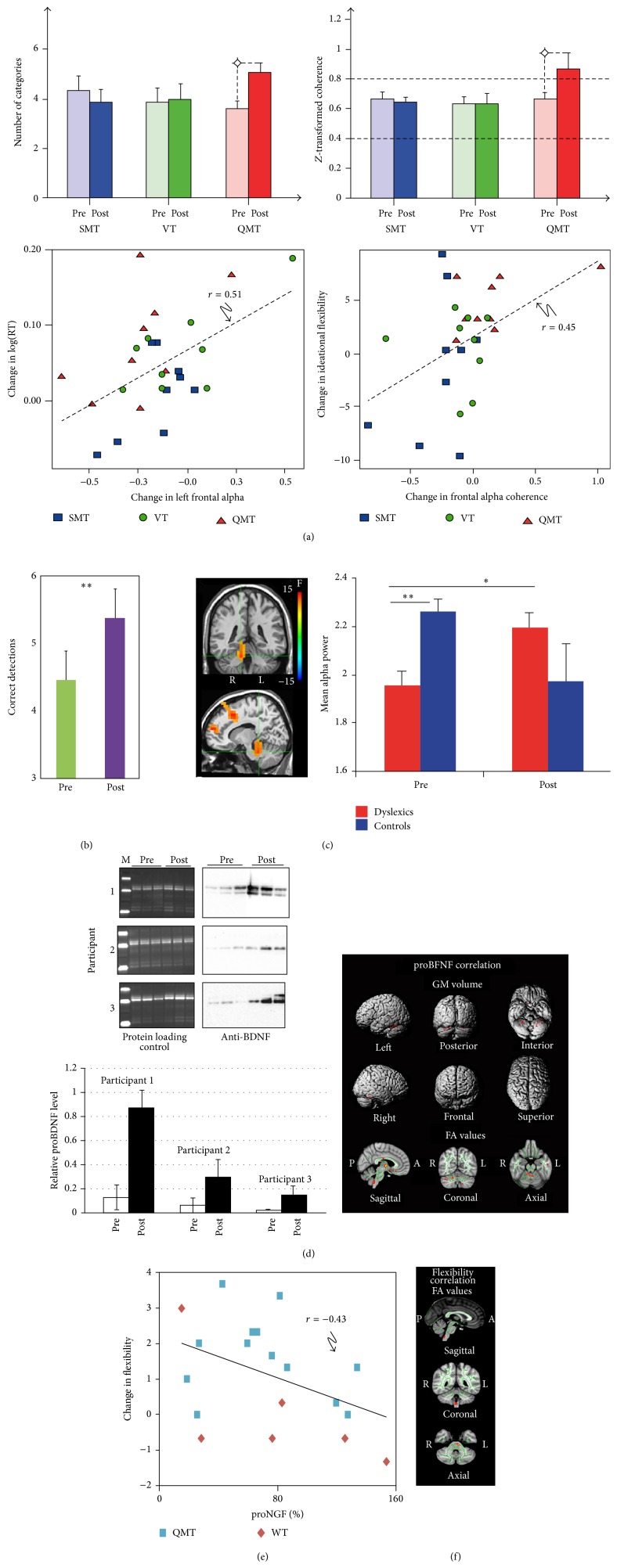
(a) Upper panel: change in ideational flexibility and* z*-transformed alpha coherence as a function of Group and Training (^*∗*^ *p* < 0.05). Lower panel: correlation between change in frontal alpha activity and cognitive change following a session of QMT. Lower panel: correlations between change in frontal alpha power and log(RT) and frontal alpha coherence and ideational flexibility (*r* = 0.51, 0.45, *p* < 0.01, *n* = 27, resp.) [65]. (b) Spatial cognition. Pre-post difference in performance on the HFT task measured by the number of correct detections (mean ± SEM, ^*∗∗*^ *p* < 0.005) [64]. (c) Changes in alpha power. Left panel: significant clusters resulting from the group (dyslexics, controls) by training (pre, post) interaction. The focus point (green cross) is positioned in the right culmen. Right panel: the bar graph shows cerebellar alpha power as a function of Group and Training (^*∗*^ *p* = 0.01; ^*∗∗*^ *p* = 0.001) [66]. (d) Changes in proBDNF and the cerebellum following 12 weeks of daily QMT practice. Left panel: Western blot analysis of proBDNF level. Histograms represent the average of the triplicate proBDNF values. Right panel: regions of GM volume and FA values positively correlated with proBDFN [67]. (e) Significant correlation between change in ideational flexibility and proNGF (beyond groups). Change in ideational flexibility, calculated by the subtraction of pre- from posttraining, was negatively correlated with the change in proNGF [68]. (f) Structural changes and creativity. A positive correlation (*p* < 0.005) between change in ideational flexibility and QMT-induced FA changes, mostly located in the middle cerebellar peduncles [69].

**Figure 4:**
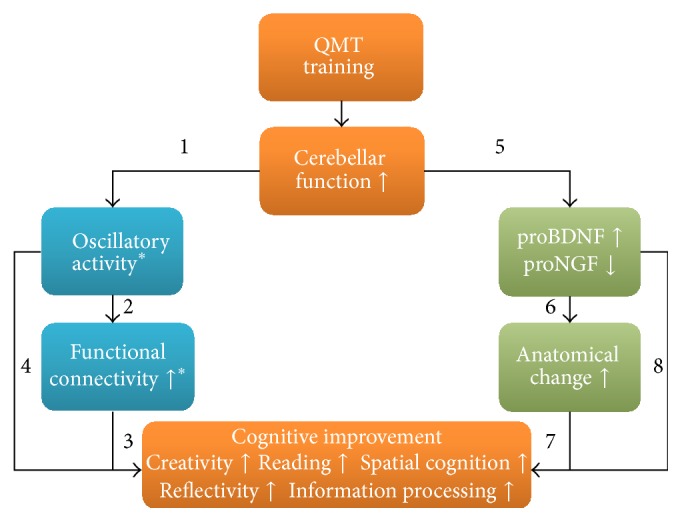
QMT-induced effects on cerebellar-cognition relationship. (*∗*) the alterations in the alpha activity and functional connectivity depending on population. While increased frontal and cerebellar alpha power were found in dyslexic adults following QMT [66], the opposite pattern, namely, decreased power, was observed in normal readers [64, 66]. In addition, while functional connectivity generally increased in females [64, 65] an opposite pattern was observed in males [64].

In the attempt to uncover the underlying mediating electrophysiological mechanism for movement-induced cognitive change, the effects of QMT were first examined in terms of EEG alpha power and coherence. In addition, changes in creativity and reaction time were examined [65]. Briefly, creativity was studied by means of the Alternate Uses Task (AU), measuring ideational fluency and ideational flexibility. In order to determine whether training-induced changes were driven by the cognitive or the motor aspects of the training, we used two control groups:* Verbal Training* (VT, identical cognitive training with verbal response) and* Simple Motor Training* (SMT, similar motor training with reduced choice requirements). Twenty-seven participants were randomly assigned to one of the groups, all practicing one session of 7 minutes. While reaction time was faster in both motor groups, only QMT enhanced interhemispheric and intrahemispheric alpha coherence, and increased ideational flexibility, which was not the case for either the SMT or VT groups (see Figure 3(a)). The different steps in model described in Figure 1 are further examined and detailed in Figure 4 describing specific QMT-induced changes.

Together these results indicate that the combination of the motor and cognitive aspects embedded in the QMT is important for increasing ideational flexibility and functional connectivity. In addition, a general decrease in alpha power was found following training, which may be related to the fact that decreased frontal alpha power is related to motor planning occurring at the contralateral side to the movement [77]. Faster reaction time was correlated with decreased frontal alpha power, providing initial support to arrows 2 and 4 in Figure 4, supporting the suggested link between training-induced alterations in alpha power and information processing. Importantly, increased frontal alpha coherence was significantly correlated with enhanced ideational flexibility, providing initial support to arrow 3 in Figure 4, supporting the suggested link between increased functional connectivity in the alpha range and enhanced creativity [65]. See Figure 3(a).

#### 2.1.2. Spatial Performance, Reflectivity, and Electrophysiology

Another study examined the possible effect of QMT on spatial cognition and reflectivity, employing the Hidden Figures Test (HFT) [78]. HFT required locating a simple figure embedded within a complex figure. Spatial performance was measured by correct answers, whereas reflectivity, namely, the ability to exercise introspection by examining one's conscious thoughts and feelings, resulting in the inhibition of habitual thought and behavior, was interpolated from correct answers and reaction time (for details, see [64]). In this study, the participants (*n* = 24, females) were randomly allocated to either QMT, SMT, or VT.

One session of QMT was found to significantly improve HFT performance, compared to SMT and VT groups, demonstrating that QMT improves HFT performance above the pre-post expected learning. This study generally showed that QMT induces enhanced reflectivity and spatial performance. However, the possible mediating mechanism was not investigated. Thus, in another study reported in the same article we examined both reflectivity and electrophysiology [64]. In this study, thirty-seven participants (20 males) were examined for gender-related differences. While QMT-induced reflectivity and spatial cognition was reported in both genders (Figure 3(b)), a gender-dependent difference in functional connectivity was observed: while theta (4–7 Hz) and alpha intrahemispheric coherence was enhanced in females, the opposite pattern was found in males. These results are consistent with the idea that neural efficiency in males is reflected in local cortical oscillations, whereas neural efficiency in females is manifested in the functional coupling of several brain areas, as assessed by EEG coherence [79]. In the next section, we will examine the long-term effects on anatomical connectivity and neurotrophic level. These will be further linked to cognitive changes found following QMT.

### 2.2. Long-Term QMT-Induced Effects

Next, we turned to investigate the direct involvement of the cerebellum in cognitive improvement. To this end, we used MEG, molecular signals and MRI, in conjunction with various cognitive tests, including reading and creativity. Here, we tested long-term effects: 1–3 months of daily QMT sessions, to enable training-induced brain functional and structural modulation and reorganization.

#### 2.2.1. QMT-Induced Effects on Reading and Cerebellar Electrophysiology

Due to the important role of the cerebellum and cerebellar alpha power in voluntary action [8–10] and its involvement in language and reading [33, 34, 38], it was hypothesized that QMT will increase cerebellar alpha power, which would in turn serve to improve reading. Thus, in the next study, the potential interactions between sensorimotor and reading systems and the role of the cerebellum oscillatory activity as a mediator [75, 76] were explored [66]. QMT was completed for a period of one month, in order to test its efficacy in inducing local and long-distance alpha oscillations. MEG was used due to its ability to localize the source of signals stemming from the cerebellum, thus enabling a direct investigation of the cerebellar role in reading improvement. QMT-induced alterations in alpha power and coherence were examined in a group (*n* = 12) of adult dyslexics and matched controls (*n* = 10 normal readers) in addition to reading performance using a one-minute reading task.

The results demonstrate that one month of intensive QMT resulted in improved performance on the speeded reading task in both the dyslexic and control groups. See Figure 3(c). While the dyslexic group suffered from decreased cerebellar alpha power at baseline compared to the normal readers, they showed a significant increase in cerebellar oscillatory alpha power following a month of QMT training [66], located in the culmen, a region which has been previously reported to be related to language processing [33, 34]. As regards connectivity, interhemispheric alpha coherence was higher in the dyslexic group compared to the control group across both time points, suggesting that increased alpha coherence may reflect a compensation mechanism ([80]; and for additional details see [66]). These findings suggested that the combination of motor and language training embedded in QMT increases cerebellar oscillatory activity in dyslexics and improves reading performance in both groups. These results support the hypothesis that the cerebellum plays a role in skilled reading and begin to unravel the underlying mechanisms that mediate cerebellar contribution in cognitive and neuronal augmentation, supporting arrows 1 and 2 in Figure 4.

In addition, while there were no significant differences between the groups in these frontal areas prior to training, the healthy control group showed a significant decrease in alpha power in the left medial frontal gyrus (MFG), right superior frontal gyrus (SFG), and supplementary motor area (SMA) following 4 weeks of daily QMT. This is similar to the effects of a session of QMT [65]. On the other hand, the opposite pattern was observed in the dyslexic group in which alpha power increased in the right SFG.

#### 2.2.2. QMT-Induced Effects on BDNF and Anatomy

As stated above, cerebellar changes are related to neurotrophic level, and specifically to BDNF [53, 54], reporting variations following training [58–62]. As the multidisciplinary studies combining the examination of training-induced neuronal and molecular changes in humans are scarce, the relationship between training-induced functional, anatomical, molecular, and cognitive change has yet to be established.

Consequently, in order to examine this relationship, proBDNF level was investigated following 3 months of daily QMT practice [67]. Briefly, a pilot MRI longitudinal study was conducted, which was designed to identify the possible link between anatomical and molecular effects of a long-term QMT. Structural high-resolution 3D T1-weighted (sMRI) and diffusion tensor imaging (DTI) data were acquired for three healthy female volunteers. For DTI, fractional anisotropy (FA) value, a marker of white matter (WM) integrity was used. Salivary BDNF was examined using Western blot analysis. The bands corresponding to proBDNF were quantified with Image Lab software and normalized to the most intense band visible on the membrane in the protein loading control. Following QMT, a significant GM volume increase in the cerebellum was found, especially in the culmen, which as stated has been previously reported to be related to language [33, 34], as well as in the right thalamus and limbic lobe. In addition, FA increases were mainly located in the corpus callosum, anterior thalamic radiations, corticospinal tracts, and cerebellar peduncles through which the cerebellum connects to the frontal cortex and other brain regions were reported. The correlation analysis revealed positive correlations between proBDNF values and GM and FA maps located in the cerebellum and cerebellar peduncle, respectively. See Figure 3(d).

This study, albeit exploratory in nature, provided important preliminary support to the relationship between sensorimotor training-induced anatomical and molecular changes (arrows 4 and 5 in Figure 4); it may shed light on the relationship between cerebellar activation and increased BDNF.

#### 2.2.3. QMT-Induced Effects on NGF and Creativity

The previous study did not examine a direct or correlational relation between change in molecular and anatomical function with cognitive change. To study such a possible relationship between molecular and cognitive change, QMT-induced neurotrophic (NGF) change and creativity were examined following 4 weeks of daily training in two interrelated studies.

In the first study [68], the effects of motor training on NGF and creativity were measured, comparing QMT and walking training (WT) in healthy adults. Creativity was measured utilizing the AU task. In contrast with the WT, QMT resulted in increased creativity, emphasizing the importance of combining cognitive control with motor training. Importantly, the change in creativity negatively correlated with the change in proNGF levels (see Figure 3(e)), supporting the negative relationship between neurotrophic factors and cognition (represented by arrow 8 in Figure 4).

In the second study, QMT-induced effects on creativity (measured by the Torrance Test of Creative Thinking (TTCT) task) and additional metacognitive functions were examined in children, using a nonintervention group as control. A total of twenty healthy children participated in this study. Following a month, 12 healthy children finished the training. Similar to the first study, a negative correlation of proNGF with QMT-induced creativity was found. Decreased proNGF further correlated with improved working memory updating and planning ability, as measured by the random number generation (RNG) and the Cognitive Assessment System (CAS) tasks, respectively. To sum up, creativity increased following a month of daily QMT practice, which further correlated with decreased proNGF level [68], supporting the negative relationship between proNGF and various cognitive functions, such as creativity [68] (arrow 6 in Figure 4).

A recent study demonstrated that increased creativity as measured by the AU task was correlated with increased cerebellar volume (under review). More specifically, a positive correlation was found between QMT-induced increased flexibility and GM increment in the right cerebellum (culmen) and the superior frontal gyrus (SFG). In addition, a positive correlation was found between increased flexibility and FA changes, mostly located in the middle cerebellar peduncles (Figure 3(f)), providing support for arrow 7 in Figure 4.

## 3. Summary and Conclusions

In our model, we propose a novel multimodal approach uniting training-induced electrophysiological changes, anatomical and neurotrophic changes, and suggest the cerebellar slow rhythm oscillations as the mediating mechanism allowing these effects to occur (Figure 4). Based on the literature, our model suggests that the importance of training-induced cerebellar changes for cognitive improvement may be based at least partly on two interrelated pathways the first being alterations in oscillatory activity, leading to changes in functional connectivity [35–37], and the second which takes longer periods to occur is mediated by neurotrophic factors, leading to anatomical changes [48].

While it has recently been suggested that sensorimotor training can result in neurotrophic-dependent changes in connectivity resulting in cognitive improvement [55, 56], the novelty of our current approach, presented in Figure 1, is in bringing forth a multimodal approach, linking cerebellum and cognition via electrophysiological fast occurring changes, anatomical and neurotrophic changes which require longer time to occur, and introduces cerebellar alpha oscillations as the mediating mechanism allowing these effects to occur. Together, although preliminary in their nature, the results presented in the current review support the hypothesis that sensorimotor training promotes alteration in cerebellar alpha oscillations which may mediate improved cognitive performance, such as enhanced creativity, faster information processing, reading, and reflectivity [64–66]. At the same time, sensorimotor training leads to anatomical and molecular changes, which are further supportive of cognitive change [67, 68].

In terms of functional connectivity, QMT practice increases inter- and intrahemispheric coherence in healthy adults [65]. Similar to studies demonstrating that frontal EEG coherence during tasks appears to be associated with improved cognitive functioning and creativity [81, 82], increased QMT-induced frontal connectivity is related to enhanced ideational flexibility [65]. Long-term QMT practice appears to further increase structural connectivity and cerebellar volume and altered cerebellar alpha activity [66]. QMT-induced anatomical changes in the cerebellum are correlated with increased BDNF level [67]. In turn, molecular change, especially proNGF decrease, is correlated with QMT-induced cognitive improvement in both children and adults [68]. Together, these findings support our proposition that cerebellar alpha oscillations may be the electrophysiological mechanism mediating neurotrophic-dependent connectivity changes resulting from sensorimotor training.

The current results are in line with previous studies demonstrating the relationship between healthy development, cerebellar activity, and BDNF [53, 54]. The cerebellum is known to be related to interoceptive accuracy reflecting explicit awareness of bodily processes [83]. Similar to the cerebellum, BDNF has also been associated with interoceptive awareness [84]. In fact, in parallel to suffering from deficient interceptive and motor awareness, Alzheimer's patients suffer from alterations in BDNF [85, 86].

The current QMT-related results found in healthy participants demonstrate increased amounts of proBDNF following 3 months of daily QMT practice [67], in addition to decreased proNGF following the training [68]. These results appear to be symmetrical to the ones conducted on the dementia patients [85–88], supporting previous claims for the inverse directionality of change in BDNF and proNGF following training [89]. Although limited indication is available on the influence of sensorimotor training paradigms on neurotrophin levels in the cerebellum, molecular change as measured by salivary proBDNF and proNG may serve an important cost-efficient benchmarks to help guide future research on the effects and the efficacy of different sensorimotor practices in both children and adults.

An important point that is raised from the reviewed studies is that Simple Motor Training which does not include a cognitive attention element could not induce such effects on creativity, as shown for the SMT group in one study [65] and by the walking training group in another study [68]. This indicates that the involvement of an attention-demanding task is necessary in order to induce these effects [65, 68]. Even though this still has to be verified, we hypothesize that such QMT effect on attention is still mediated through the cerebellum, which may in turn be affecting executive frontal regions [16]. This claim is partly supported by the fact that QMT resulted in similar changes in alpha power in both the cerebellum and frontal areas in healthy adults [66].

Undoubtedly, the model presented here is very preliminary, and much future research is required in order to sufficiently validate all its facets. However, it provides a framework for future research and raises many scientifically testable predictions, important for guiding future directions.

## 4. Future Directions

The cerebellar deficits in development disorders may well explain the fact that, in a range of developmental disorders, such as ADHD, dyslexia, and autism, in parallel to the cognitive deficits, children suffer from deficient motor function and sensorimotor symptoms, thus having a higher probability of developing psychopathology [71, 72].

Since sensorimotor deficits are often observed in different developmental disorders, some researchers attributed their cognitive and motor deficiencies to abnormal development and functioning of the cerebellum [33–54]. In parallel to the cognitive deficits, both patients with different developmental disorders as well as neurodegeneration disease suffer from deficient sensorimotor function and emotional challenges [71, 72, 90] emphasizing the importance of efficient sensorimotor training paradigms for means of possible treatment. The examination of training-induced changes in functional and structural connectivity together with molecular change and their relations to cognition could greatly benefit from further studies in healthy and disabled populations. In order to verify whether cerebellar alpha oscillations are the underlying electrophysiological mechanism mediating change in cerebellar size, a combination of MRI and EEG should be implemented.
